# Individual differences in reward prediction error: contrasting relations between feedback-related negativity and trait measures of reward sensitivity, impulsivity and extraversion

**DOI:** 10.3389/fnhum.2014.00248

**Published:** 2014-04-28

**Authors:** Andrew J. Cooper, Éilish Duke, Alan D. Pickering, Luke D. Smillie

**Affiliations:** ^1^Department of Psychology, Goldsmiths, University of LondonLondon, UK; ^2^Melbourne School of Psychological Sciences, The University of MelbourneMelbourne, VIC, Australia

**Keywords:** extraversion, pleasure, feedback-related negativity, reward, event related potential, behavioral approach system

## Abstract

Medial-frontal negativity occurring ∼200–300 ms post-stimulus in response to motivationally salient stimuli, usually referred to as feedback-related negativity (FRN), appears to be at least partly modulated by dopaminergic-based reward prediction error (RPE) signaling. Previous research (e.g., Smillie et al., 2011) has shown that higher scores on a putatively dopaminergic-based personality trait, extraversion, were associated with a more pronounced difference wave contrasting unpredicted non-reward and unpredicted reward trials on an associative learning task. In the current study, we sought to extend this research by comparing how trait measures of reward sensitivity, impulsivity and extraversion related to the FRN using the same associative learning task. A sample of healthy adults (*N* = 38) completed a battery of personality questionnaires, before completing the associative learning task while EEG was recorded. As expected, FRN was most negative following unpredicted non-reward. A difference wave contrasting unpredicted non-reward and unpredicted reward trials was calculated. Extraversion, but not measures of impulsivity, had a significant association with this difference wave. Further, the difference wave was significantly related to a measure of anticipatory pleasure, but not consummatory pleasure. These findings provide support for the existing evidence suggesting that variation in dopaminergic functioning in brain “reward” pathways may partially underpin associations between the FRN and trait measures of extraversion and anticipatory pleasure.

Monitoring and evaluating cues in the environment for their motivational significance and reward value represents a crucial aspect of decision-making and goal-directed behavior. Cues that provide feedback on whether outcomes of actions have been better or worse than expected are critical in the updating of behavior in response to environmental demands. In particular, reinforcement learning models often train a so-called actor circuit (which associates stimuli with responses) using a teaching (reinforcement) signal sent from a critic circuit, which compares actual with predicted outcomes. Such models typically stress the temporal prediction of reinforcement, relying on so-called temporal difference learning (see Sutton and Barto, 1998, for a review). Dopaminergic (DA) projections from the ventral tegmental area (VTA) to the nucleus accumbens and the anterior cingulate cortex (ACC) appear to play a key role in signaling the degree to which events are better or worse than expected. This has been termed reward prediction error (RPE) signaling (e.g., Schultz, 1998, 2007), and this DA-based RPE signaling function has been widely argued to be a central part of the biological underpinning of reinforcement learning mechanisms within actor-critic (e.g., Houk et al., 1995), temporal difference (e.g., Montague et al., 1996), and other (e.g., Brown et al., 1999) models.

The ACC appears to play an important integrative role as a recipient of RPE signals, using this information to assign reward value to cues, evaluate the effect of previous actions and select subsequent actions, amongst other functions (Gehring and Willoughby, 2002; Holroyd and Coles, 2002; Paus, 2001). The DA mediated RPE signal has been associated with a negative deflection in the event related potential (ERP) approximately 200–300 ms after the presentation of motivationally salient feedback, and is largest in magnitude over medial-frontal brain areas (Holroyd and Coles, 2002; Nieuwenhuis et al., 2004). Although originally identified with an earlier response to error commission (often termed error-related negativity), the negative deflection 200–300 ms post-stimulus has been observed in the absence of any overt choice or response, and so has been termed feedback-related negativity (FRN; Yeung et al., 2005). Studies using source localization analyses and functional imaging have suggested the ACC is the source of the FRN (Gehring and Willoughby, 2002; Ruchsow et al., 2002; Holroyd et al., 2004; Martin et al., 2009). Holroyd and colleagues have suggested that the FRN is modulated by phasic DA responses to unpredicted rewards and unpredicted non-rewards that serve as inputs to the ACC. In this way, the FRN has been linked with the generation of RPE signals that are transmitted via ascending dopaminergic pathways; phasic decreases in DA activity in response to unpredicted non-rewards result in a more negative FRN, while increases in phasic DA activity result in a less negative FRN (Holroyd and Coles, 2002; Nieuwenhuis et al., 2004; Holroyd and Krigolson, 2007).

On the bases outlined above, the difference in the magnitude of FRN should be larger when comparing unpredicted rewards and unpredicted non-rewards (i.e., the calculation of a difference wave), compared to the difference in FRN magnitude between predicted rewards and non-rewards. In other words, we can potentially characterize the difference in FRN magnitude for unpredicted reward and unpredicted non-reward trials as an index of RPE signaling. The existing empirical evidence has generally supported an inverse relation between the likelihood of outcome and the magnitude of an FRN difference wave between unpredicted reward and unpredicted non-reward trials (Walsh and Anderson, 2012). For example, Potts et al. (2006) examined ERPs after participants had completed a passive associative learning task that manipulated the likelihood of reward and non-reward. They showed that FRN was most negative for unpredicted non-reward trials and least negative for unpredicted reward trials in the expected time window (i.e., 200–300 ms post stimulus presentation). Notably, FRN was elicited in the absence of any requirement for a behavioral response to the task stimuli, suggesting that FRN can reflect feedback monitoring in a general sense and is not necessarily a response-locked deflection.

The field of personality neuroscience seeks to identify the neurobiological mechanisms, along with the key operational parameters of these mechanisms, that contribute to the long-term patterning of affect, behavior and cognition (DeYoung, 2010). There has long been recognition that individual differences in the sensitivity of brain systems involved in the processing of reward contribute to variation in higher order personality traits (Gray, 1973; Depue and Collins, 1999; Pickering and Gray, 1999). This has often been considered in the framework of what has been termed the Behavioral Approach System or Behavioral Activation System (BAS; Pickering and Smillie, 2008). There has been less consensus, however, on the personality trait/s that might best reflect BAS functioning. One candidate trait is extraversion (Depue and Collins, 1999; Smillie, 2013), a trait associated with positive affect, behavioral approach and agency (Wilt and Revelle, 2009). There has also been a focus on impulsivity and/or sensation-seeking, and other traits associated with anti-social behavior (Zuckerman, 1984; Pickering, 2004). This latter effort has been complicated by the recognition that factor analyses of impulsivity self-report scales show a multidimensional structure (e.g., Whiteside and Lynam, 2001; Cyders and Coskunpinar, 2011). Further still, researchers have developed novel scales to measure theory-driven conceptualizations of dispositional BAS functioning; these have also typically been multidimensional in their factorial structure, breaking down in to what might be broadly termed reward sensitivity and impulsivity traits. An example along these lines would be the Carver and White (1994) BIS/BAS scales; these scales have three BAS factors, reward responsiveness, drive and fun-seeking. More generally, factor analyses of multiple self-report “BAS-related” scales typically used in this research show a multi-factorial structure; these factors have often been labeled reward drive (or reward sensitivity) and rash impulsiveness (Dawe and Loxton, 2004; Cooper et al., 2008).

As there is now substantial empirical support for FRN as an index of DA RPE signaling, it serves as a useful tool for evaluating personality traits that have a putative basis in DA functioning. For instance, Smillie et al. (2011) used the same associative learning task reported by Potts et al. (2006; see earlier) to examine the association between FRN and individual differences on a trait measure of extraversion, assessed using the Eysenck Personality Questionnaire—Revised (EPQ-R; Eysenck and Eysenck, 1991). The study used an extreme groups design, including individuals exceeding 1 SD unit above or below the mean for extraversion. Each trial in the task involved the sequential presentation of two images, each of which was either a gold bar or a lemon. For 40% of trials there was a sequence of two gold bars followed by a monetary reward (predicted reward), while for another 40% of trials there was a sequence of two lemons followed by no reward (predicted non-reward). In addition, on 10% of trials a gold bar was followed by a lemon and no reward was delivered (unpredicted non-reward), while on the remaining 10% of trials a lemon was followed by a gold bar and a reward was delivered (unpredicted reward). Participants were not required to make any behavioral responses as part of the task, and simply observed the monetary outcomes. The results replicated findings by Potts et al. (2006), with FRN being significantly larger for unpredicted non-reward trials compared to unpredicted reward trials. Furthermore, Smillie et al. (2011) found that the difference in FRN for unpredicted non-reward and unpredicted reward trial types was larger for Extraverts, such that FRN was more negative following unpredicted non-reward and less negative following unpredicted reward. A subset of these participants had provided a DNA sample for a separate study, and so FRN was also examined in relation to the dopaminergic-related gene polymorphism DRD2/ANKK1. The results showed that those carrying at least one copy of the A1 allele had a larger difference wave contrasting unpredicted non-reward and reward trials, although this difference failed to reach formal statistical significance.

Prior to Smillie et al. (2011), a number of studies had reported associations between error and feedback-related ERP components and personality traits related to reward and punishment sensitivity (e.g., Boksem et al., 2006, 2008; Balconi and Crivelli, 2010; Tops and Boksem, 2010). For example, Boksem et al. (2006) related scores on the Carver and White (1994) BIS/BAS scales with ERP responses to errors on the Eriksen Flanker Task. They found that BIS scores were positively correlated with error-related negativity 50–100 ms post-stimulus response, with BAS scores unrelated to this component. Conversely, a later error positivity deflection was significantly and positively related to BAS scores, largely driven by the fun-seeking subscale, but was not significantly related to BIS scores. Recently, several other studies have examined relations between FRN and BAS-related traits more specifically. Lange et al. (2012) examined the relations between scores on the BIS/BAS scales and FRN in response to a two-choice task that manipulated reward expectation. They found that FRN was significantly more negative following unpredicted non-reward in the extinction phase of the task for individuals higher on the BAS scale, as they predicted. Conversely, individuals higher on the BIS scale showed a significantly less negative FRN in relation to unpredicted non-reward. Notably, an aggregated BAS scale score was used in this analysis, so it is unclear how the different BAS facets related to the FRN in this case. It leaves open the possibility that different facets of the BAS, in this case reward responsiveness, drive and fun-seeking, may have diverging associations with FRN magnitude. More recently, Bress and Hajcak (2013) examined FRN responses to a gambling task, and associations with a self-report measure of reward responsiveness (RR; Van den Berg et al., 2010) and a signal detection behavioral task designed to assess bias towards reward. This self-report measure of RR shares some items with the reward responsiveness and drive scales from the BIS/BAS scales; an inspection of the item content in this new RR scale arguably suggests that it reflects behavioral approach and agency, rather than the enjoyment or consummation of reward. Bress and Hajcak (2013) found that self-reported RR using this new scale significantly correlated with the difference between FRN response to gains and losses, such that those higher on RR had a larger magnitude difference wave.

In sum, there is a small but growing body of research suggesting that individual differences in BAS-related personality traits are related to the magnitude of FRN. To date, however, these studies have tended to either examine individual BAS traits in isolation, or have used aggregated BAS scales, potentially obscuring important dissociations in the relations between different BAS traits and FRN magnitude.

Another potential issue with the previous research on FRN and BAS-related traits, and arguably an issue in the reward processing and personality literature more generally (see Smillie, 2013), is an under-appreciation of the distinction between different aspects of reward processing, and what these aspects might mean for individual differences in BAS-related personality traits. For example, in the addiction literature a distinction has been made between reward “wanting”, referring to the motivated approach of and feelings of desire for reward, with a putative basis in mesolimbic dopaminergic functioning, and reward “liking”, referring to feelings of enjoyment or satisfaction upon reward consummation, with a putative basis in forebrain opioid circuitry (Berridge et al., 2009). Efforts in other areas of clinical psychology have highlighted the potential value in dissociating the motivational and consummatory components of reward processing, particularly in relation to the reward processing deficiencies often seen in depression and schizophrenia (Treadway and Zald, 2011, 2013). Gard et al. (2006) have developed a self-report questionnaire, the Temporal Experience of Pleasure Scale (TEPS), which sought to measure trait individual differences in anticipatory pleasure (TEPS-ANT) and consummatory pleasure (TEPS-CON). While there has been some evidence to suggest dissociations between the two scales in relation to reward processing deficits in schizophrenic patients (Gard et al., 2007), the evidence on this front is mixed (Strauss et al., 2011). More generally, further research is needed to validate the psychometric distinction between these constructs.

Our aim in this study was to extend the existing data by examining how a broad array of BAS-related personality measures relate to FRN magnitude, using the same associative learning task reported in Smillie et al. (2011). The personality inventories we included cover constructs that have currently or previously been thought to at least partly reflect variation in the BAS, and so might be considered candidates for relating to neural indices of dopaminergic functioning. These include measures of extraversion, impulsivity and reward sensitivity/anhedonia. We were particularly interested in examining the TEPS in this context, the subscales of which potentially capture the distinction between reward wanting and liking. Given the putative basis of the approach or anticipatory element of reward processing in dopaminergic functioning, we would predict that the TEPS-ANT scale would significantly vary with FRN, but we would expect to see no significant association between the TEPS-CON scale and FRN. Similarly, with the BAS scales from the Carver and White (1994) scales, we would predict that FRN would be significantly predicted by the drive subscale, but not by reward responsiveness (the item content of which appears to capture consummatory aspects of reward processing) or fun-seeking (which appears to measure impulsivity; Smillie et al., 2006). Our measure of extraversion in the current study was the same as that used in Smillie et al. (2011); the EPQ-R, and so we would expect that this measure would also relate significantly with FRN, but we would predict no association with the psychoticism scale from the EPQ-R (as this is where impulsivity lies within the Eysenckian “giant three” framework). Measures used in the clinical assessment of anhedonia included in the current study, the Snaith-Hamilton Pleasure Scale and the anhedonia subscale from the Beck Depression Inventory, both tend to have item content reflective of the consummatory aspects of reward processing, and so we would not expect to see a significant association with FRN for these measures.

## Methods

### Participants

Thirty-eight right handed individuals aged between 19 and 42 years (*M* = 24.39, SD = 4.76) participated in this study in exchange for cash (£15); 20 of these participants were male (52.6%). Participants were largely recruited from among students at Goldsmiths, University of London, UK. No participants reported a personal history of psychiatric illness and all participants had normal or corrected-to-normal vision. Participants were recruited via leaflets and social networking sites. All participants provided written consent to take part in the study. The experimental procedure, including EEG set-up, was outlined prior to the start of the experiment and participants were given the opportunity to ask questions and were made aware that they could withdraw participation at any point during the study. These procedures were approved by the Goldsmiths Department of Psychology Ethics Committee.

### Measures

After completion of consent and information forms, and prior to the EEG recording and completion of the experimental task, participants completed a battery of personality measures comprised of the following:

#### Temporal experience of pleasure scale (TEPS)

The TEPS (Gard et al., 2006) is an 18-item questionnaire designed to measure individual differences in anticipatory pleasure (TEPS-ANT; 10 items) and consummatory pleasure (TEPS-CON; 8 items). The TEPS-ANT subscale measures feelings of pleasure associated with anticipation and eagerness for upcoming events e.g., “*When something exciting is coming up in my life, I really look forward to it*”. The TEPS-CON subscale measures feelings of pleasure associated with the consumption and savoring of current rewarding events e.g., “*A hot cup of coffee or tea on a cold morning is very satisfying to me*”. Participants indicated their agreement with the 18 statements using a 6-point Likert-type scale ranging from *strongly disagree* to *strongly agree*. Individual item scores were summed for each subscale, such that high scores equate to stronger feelings of pleasure. In the current study, Cronbach’s α was 0.72 for TEPS-ANT and 0.50 for TEPS-CON. The somewhat low reliability estimate for the TEPS-CON subscale is consistent with some previous studies that have used this questionnaire, albeit with a Chinese language version (Chan et al., 2012) and an English language version used with patients diagnosed with schizophrenia (Buck and Lysaker, 2013).

#### The BIS/BAS scales

The Carver and White (1994) BIS/BAS Scales are a measure comprising a BIS scale (7 items) and three BAS scales: reward responsiveness (5 items), drive (4 items) and fun-seeking (4 items). Each item was answered using a four-point Likert scale, ranging from 1 (“*very false for me”*) to 4 (“*very true for me”*). Previous research has shown the scales have satisfactory internal reliability and construct validity (Carver and White, 1994; Gomez et al., 2005). Item scores for each subscale were summed, with higher scores equating to higher approach and inhibition. Cronbach’s α-values in the current study for reward responsiveness, drive, fun-seeking and BIS were 0.69, 0.78, 0.60, and 0.76, respectively.

#### The eysenck personality questionnaire—revised (EPQ-R)

The EPQ-R (Eysenck and Eysenck, 1991) is a widely used measure of personality that provides scores for extraversion (23 items), neuroticism (24 items), and psychoticism (32 items). The extraversion subscale includes items that reflect behavioral approach and agency, while the psychoticism subscale includes items that reflect impulsive and anti-social behavior. The neuroticism subscale includes items that reflect negative affective states and emotional instability. Respondents indicated their agreement with each statement using a dichotomous yes/no response format. Item scores for each subscale were summed, with higher scores equating to higher levels of the respective trait. The EPQ-R has been used extensively in past research, and has been shown to have good reliability and validity. In the current study, Cronbach’s α-values for extraversion, neuroticism, and psychoticism were 0.74, 0.86, and 0.76, respectively.

#### The beck depression inventory (BDI)

The Beck Depression Inventory–II (BDI-II; Beck et al., 1996b) is a widely-used self-report measure assessing the severity of depressive symptoms over the previous 2 weeks, with good reported reliability and validity (Beck et al., 1996a). Items 4 (“*satisfaction with things”*), 12 (“*interest in other people”*), 15 (“*effort in doing things”*), and 21 (“*interest in sex”*) can comprise an anhedonia subscale (e.g., Pizzagalli et al., 2005), and this subscale was also examined separately in the current study. Cronbach’s α-values for the total BDI scale and the anhedonia subscale in the current study were 0.85 and 0.38, respectively. All participants in this sample had the same response to item 21 (i.e., no change in interest in sex), therefore this item was not included in the calculation of Cronbach’s α for the BDI total and anhedonia subscales.

#### Snaith-Hamilton pleasure scale (SHPS)

The Snaith-Hamilton Pleasure Scale (SHPS; Snaith et al., 1995) is a 14-item self-report measure of the pleasure felt when engaging in various everyday activities (e.g., “*I would enjoy a warm bath or refreshing shower”*). Respondents indicated the degree to which they agreed with each statement using a four-point scale (“*Strongly Disagree”*, “*Disagree”*, “*Agree”* and “*Strongly Agree”*). All statements are positively worded. To derive a total score, either of the “disagree” responses to an item is given 1 point, and either of the “agree” responses is given 0 points; thus, total scores can range from 0–14, with higher scores indicative of higher levels of anhedonia. Cronbach’s α for the SHPS in the current study was 0.68.

### Task design and procedure

Following completion of the personality measures, participants were seated in a noise-shielded room in front of the computer screen showing the experimental task and the EEG recording procedure was initiated, as outlined below. Once the EEG equipment had been fitted, participants were given instructions for the task. The experimental task used in the current study was the same as that described in Smillie et al. (2011), which itself had been based on an earlier task used by Potts et al. (2006). The task was presented to participants as being similar to a “fruit machine” used in gambling venues in the UK (often called a “slot machine” or “poker machine” outside the UK). The task used a passive S1-S2 randomized-block design, with two within-subject factors representing the differences in trial-type: reward *vs*. non-reward, and predicted *vs*. unpredicted. S1 and S2 comprised images of either a gold bar or a lemon. Participants were instructed to simply observe the trials on the screen and attend to the outcome of each trial, and that they did not need to make any overt actions in response to the presentations.

Each trial sequence began with a fixation point (300 ms), followed by the presentation of S1 (500 ms), a second fixation point (300 ms), presentation of S2 (500 ms), and then feedback in the form of a numeric representation of the trial and cumulative earnings (600 ms). To help minimize blink artifacts, a “*blink now”* message appeared on the screen at the end of each trial as part of an irregular inter-trial interval (2000–3600 ms), and participants were encouraged at the beginning of the task to restrict blinking to this period if possible.

Participants completed 30 practice trials to ensure that they understood the task. They subsequently received a total of 480 experimental trials (8 blocks with 60 trials per block), which were separated by rest periods. On 40% of trials S1 and S2 were gold bars and participants received a reward (£0.50) (predicted reward; 192 trials). On another 40% of trials S1 and S2 were lemons, and participants received no reward (predicted non-reward; 192 trials). On 10% of trials S1 was a gold bar and S2 was a lemon, and participants received no reward (unpredicted non-reward; 48 trials). Conversely, on the remaining 10% of trials S1 was a lemon and S2 was a gold bar, and a reward (£0.50) was received (unpredicted reward; 48 trials). Cumulative “winnings” from each trial were reset between blocks, and participants were told that they would be paid their “winnings” from the highest-paying block (which was fixed at £15 for all participants). After completion of the task, the EEG equipment was removed and participants were debriefed on the aims of the study.

### EEG recording and analysis

Continuous EEG data were acquired from 64 active Ag/AgCl electrode channels placed in accordance with the extended 10–20 system using Easycap® elastic electrode caps. In order to detect eye movements [electrooculogram (EOG)], two electrodes were placed on the sub- and supra-orbit of the right eye to monitor vertical eye movements, and an additional two electrodes recorded the horizontal EOG from the external canthi of both left- and right eyes. The active electrode system did not require impedance measurements. Data were amplified using a BioSemiActiveTwo® amplifier. To help ensure that the recorded data was of a high standard, the experimenter continuously monitored the incoming EEG data, and participant attention and body movements were observed via a closed circuit video camera. All data were sampled at 512 Hz, and further filtered offline using a 0.1–100 Hz bandpass filter. An average reference was applied to the data. The data was segmented in to 500 ms epochs, beginning 100 ms before S2 onset and finishing 400 ms post S2-onset. Individual epochs were extracted for the onset of the different trial types (unpredicted reward, unpredicted non-reward, predicted reward, predicted non-reward), and these were time-locked to the S2 onset.

Artifacts were automatically detected according to a maximum/minimum voltage criterion (±70 µV on target frontal channels and EOG channels), and then kept or rejected after visual inspection. Following artifact rejection, there was a mean of 36.51 (SD = 9.86) and 36.70 (SD = 9.84) trials available for subsequent analysis for unpredicted reward and unpredicted non-reward trials, respectively. For the more common trial types, there was a mean of 145.84 (SD = 37.36) and 146.46 (SD = 37.95) trials available for subsequent analyses for the predicted reward and predicted non-reward trial types, respectively. There were no significant correlations between the number of trials after artifact rejection for each of the trial types and scores on any of the personality variables. The FRN was averaged across six medial-frontal sites (F1, F2, Fz, FC1, FC2, and FCz), and a grand average was calculated for each participant for each of the four conditions.

In line with the approach by Smillie et al. (2011), we exported the mean ERP amplitude during a time window of 200–300 ms post S2-onset for analysis. To provide alternative estimates, we also extracted data from (a) the six medial-frontal electrode sites mentioned above, but using a longer time window post S2-onset (e.g., 200–350 ms); (b) from the same electrode sites using the difference in magnitude of the N2a and P3 peaks; and (c) from the single medial-frontal channel Fz. All of these alternate indices correlated >0.95 with our index based on the six medial-frontal sites over the 200–300 ms window post S2-onset on our key outcome variable (e.g., the averaged unpredicted reward-unpredicted non-reward difference wave), and associations with the personality variables across these alternate indices were very similar.

## Results

### Personality measures

Means and standard deviations for the personality measures and the correlations between these measures are shown in Table 1. Of note, the TEPS-ANT scale had a significant positive correlation with the BAS-reward responsiveness scale, and a substantial but non-significant positive correlation with the EPQ-extraversion scale. The TEPS-CON scale also had a significant positive correlation with the BAS-reward responsiveness scale, although of a lower magnitude than the correlation between the TEPS-ANT and the BAS-reward responsiveness scales. The TEPS-CON scale also had a significant negative correlation with the SHPS, reflecting their close (but inverse) conceptual relationship. The TEPS-ANT and TEPS-CON scales were moderately positively correlated (*r* = 0.40). Females scored significantly higher on the TEPS-ANT scale, *t*_(36)_ = −4.51, *p* < 0.0001, the BAS-reward responsiveness scale, *t*_(36)_ = −3.72, *p* = 0.001, and the BIS scale, *t*_(36)_ = −2.64, *p* = 0.012; there were no significant differences across gender for any of the other personality measures.

**Table 1:** *Correlations between the trait self-report measures and the averaged difference between the ERP response to unpredicted reward and non-reward trials*.

	RPE	EPQ P	EPQ E	EPQ N	SHPS	BAS-DR	BAS-FS	BAS-RR	BIS	BDI	BDI-AN	TEPS-ANT	TEPS-CONS
RPE	1											
EPQ P	0.19	1											
EPQ E	0.36*	0.06	1										
EPQ N	−0.15	0.07	−0.33	1									
SHPS	−0.15	0.09	−0.05	0.15	1								
BAS-DR	−0.10	0.18	0.25	−0.15	−0.11	1							
BAS-FS	0.18	0.48*	0.56**	0.08	−0.03	0.42**	1						
BAS-RR	0.22	−0.22	0.22	0.13	−0.31	0.27	0.13	1					
BIS	−0.10	−0.39	−0.24	0.53**	0.21	−0.55**	−0.27	0.22	1				
BDI	−0.24	0.44*	−0.17	0.67**	0.24	0.07	0.21	−0.21	0.02	1			
BDI-AN	−0.08	0.35	0.00	0.55**	0.43**	−0.02	0.23	−0.25	0.04	0.84**	1		
TEPS-ANT	0.39*	−0.20	0.38	−0.04	−0.27	0.01	0.05	0.59**	0.19	−0.26	−0.24	1	
TEPS-CONS	0.11	−0.18	0.05	−0.06	−0.41*	0.28	0.03	0.33*	−0.06	−0.30	−0.26	0.40*	1
Mean	1.27	5.60	15.96	10.68	0.92	10.71	12.03	16.74	20.71	5.18	1.05	45.37	37.61
SD	2.88	3.21	3.98	5.32	1.51	2.24	1.94	2.10	3.48	5.21	1.38	5.99	4.82

Note: * p < 0.05, ** p < 0.01. RPE = Reward prediction error index, EPQ P = Eysenck Personality Questionnaire—Revised Psychoticism, EPQ E = Eysenck Personality Questionnaire—Revised Extraversion, EPQ N = Eysenck Personality Questionnaire - Revised Neuroticism, SHPS = Snaith-Hamilton Pleasure Scale, BAS-DR = Behavioural Approach System—Drive, BAS-FS = Behavioural Approach System—fun-seeking, BAS-RR = Behavioural Approach System—Reward Responsiveness, BIS = Behavioural Inhibition System, BDI = Beck Depression Inventory, BDI-AN = Beck Depression Inventory Anhedonia, TEPS-ANT = Temporaral Experience of Pleasure Scale—Anticipatory, TEPS-CON = Temporal Experience of Pleasure Scale—Consummatory. n = 25 for all correlations involving the EPQ-R; n = 38 for all other correlations. EPQ-R Extraversion and RPE correlation is reported using one-tailed testing; all other correlations are reported as two-tailed tests.

### Task manipulation check

**Figure 1:**
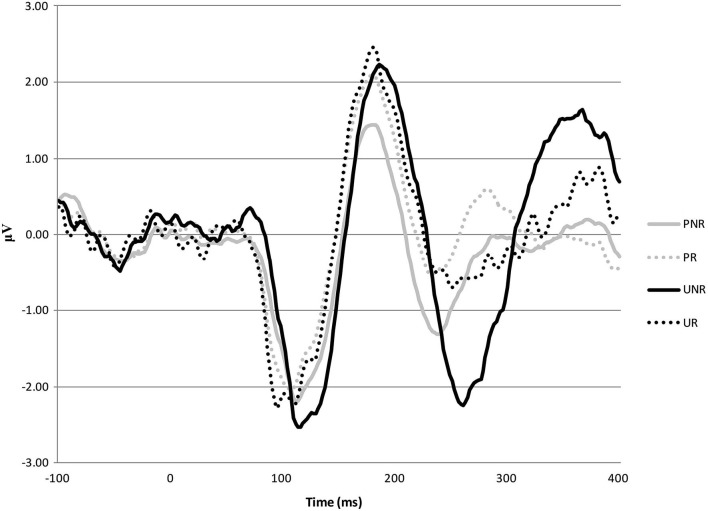
**ERP waveforms averaged across six medial-frontal sites (F1, F2, Fz, FC1, FC2, and FCz) for predicted non-reward (PNR), predicted reward (PR), unpredicted non-reward (UNR) and unpredicted reward (UR) trials across all participants**.

A 2 (predicted, unpredicted) × 2 (reward, non-reward) repeated measures ANOVA was undertaken to ensure that variation in the FRN was largely driven by ERP responses to unpredicted reward and non-reward trials. Variation in ERP response across the four trial types broadly followed the pattern seen in Smillie et al. (2011). The ANOVA showed that ERP averaged over medial-prefrontal areas was more negative for non-reward than reward trials, *F*_(1, 37)_ = 9.42, *p* = 0.004, and more negative for unpredicted than predicted trials, *F*_(1, 37)_ = 6.19, *p* = 0.017. The interaction between predicted and reward trial was not significant, *F*_(1, 37)_ = 3.07, *p* = 0.088. Several studies (e.g., Potts et al., 2006, 2010; Smillie et al., 2011) using this paradigm have found that the greatest waveform difference in this 2 × 2 design is between unpredicted reward and unpredicted non-reward trials. In the present study, the difference between these conditions was significant, *F*_(1, 37)_ = 7.40, *p* = 0.01, while the difference between the predicted reward and predicted non-reward trials was close to 0, *F*_(1, 37)_ = 0.14, *p* = 0.71. We therefore followed past practice with this paradigm and computed a difference waveform. A difference score was therefore calculated for each participant contrasting the ERP response to unpredicted reward trials and unpredicted non-reward trials (i.e., the mean amplitude of response to unpredicted reward trials minus the mean amplitude of response to unpredicted non-reward trials) as an index of RPE. This pattern of effects is shown in the ERP waveforms by trial type in Figure 1.

### Main analyses

The main analysis sought to examine whether an index of RPE related to a battery of measures assessing traits with a putative basis in dopaminergic functioning. There was no significant difference across gender for this RPE index, *t*_(36)_ = −0.45, *p* = 0.652. Firstly, we sought to replicate our earlier finding (Smillie et al., 2011), showing that EPQ-R extraversion was related to this index of RPE. We did this in a subset of participants in the current sample on whom we had EPQ-R data (*n* = 25). On the basis of our previous result with EPQ-R extraversion and this RPE index, we expected a positive correlation and so report a one-tailed test for this association with RPE (for the other personality measures we report two-tailed tests, given the more exploratory nature of this testing). The result in this sample showed a significant positive correlation between EPQ-R extraversion and the RPE index, *r* = 0.36, *p* = 0.038 (one-tailed), indicating the difference of response magnitude between unpredicted non-reward and unpredicted reward trial types tended to be larger for participants higher in extraversion, as in our previous study.

**Figure 2:**
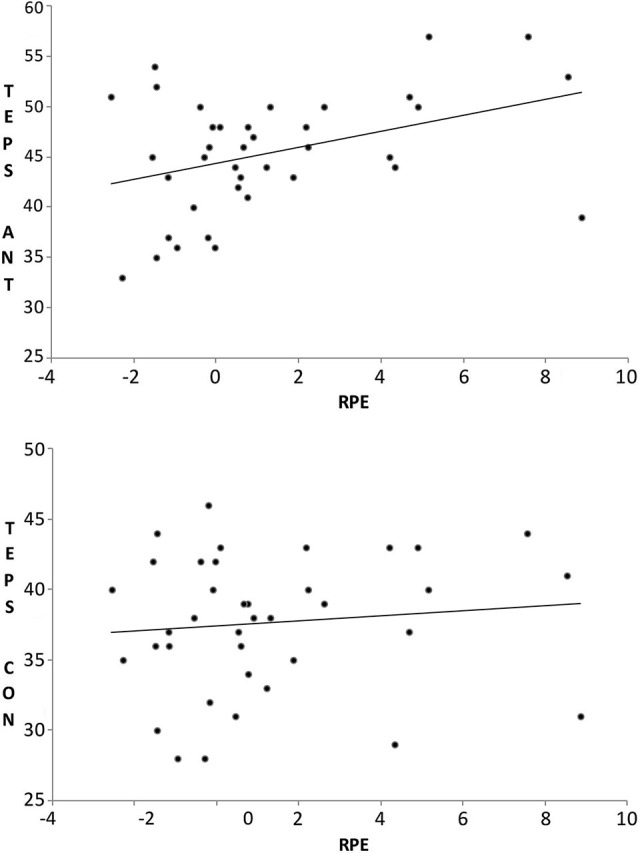
**Scatterplots showing the correlation between the Reward Prediction Error (RPE) index and the TEPS-Anticipatory (TEPS-ANT) scale and the TEPS-Consummatory (TEPS-CON) scale**.

**Figure 3:**
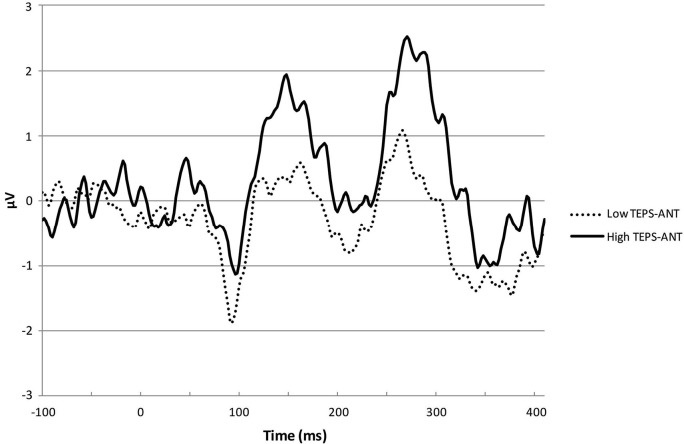
**ERP difference waveforms (unpredicted reward trials minus unpredicted non-reward trials) for individuals high and low on the TEPS-Anticipatory (TEPS-ANT) scale**.

Table 1 shows the correlations between the other trait measures used in the study and the RPE index (i.e., the averaged difference wave across unpredicted reward and unpredicted non-reward trials). As expected, the RPE index was significantly and positively correlated with the TEPS-ANT scale (*r* = 0.39), but the RPE index did not correlate significantly with the TEPS-CON scale (*r* = 0.11). A test of the difference between the two related correlation coefficients was carried out to investigate the prediction that the correlation between the TEPS-ANT and the RPE index would be significantly larger than the correlation between TEPS-CON and the RPE index. This comparison was significant, $Z_{1}^{*}=1.60$, *p* = 0.05, one-tailed $Z_{1}^{*}$ is a recommended statistic for this comparison, Steiger, 1980). This indicates the difference of FRN response magnitude between unpredicted non-reward and unpredicted reward trial types tended to be larger for participants higher on trait anticipatory, but not consummatory, pleasure. The correlation between the RPE index and the TEPS-CON should be treated with caution, however, given the low reliability of the TEPS-CON in this sample. Figure 2 shows scatterplots of these two sets of associations. A model regressing the RPE index on TEPS-ANT and TEPS-CON simultaneously showed that TEPS-ANT, β = 0.41, *t* = 2.39, *p* = 0.02, but not TEPS-CON, β = −0.05, *t* = −0.31, *p* = 0.76, was a significant predictor of the RPE index. For illustrative purposes, we split participants in to high and low TEPS-ANT and show the difference waveforms for the two groups in Figure 3 (the divergence between these waveforms at around 100–150 ms potentially reflects individual differences in error-related negativity, with which FRN has been associated).

Table 1 shows that none of the other trait self-report measures correlated significantly with the RPE index, including measures that primarily assess anhedonia or lack of pleasure (SHPS and BDI-anhedonia), negative affect more generally (BDI, BIS, and EPQ N), impulsivity (BAS-fun-seeking and EPQ-P), and, more surprisingly, the remaining BAS scales (BAS-drive and BAS-reward responsiveness).

## Discussion

The results from this study provide further support for the notion that FRN may be at least partly mediated by RPE signaling (Holroyd and Coles, 2002). Similar to Potts et al. (2006) and Smillie et al. (2011), we found that FRN was significantly more negative for unpredicted non-reward trials when compared with unpredicted reward trials, while there was no significant difference in negativity for predicted reward and predicted non-reward trial types. Further, we replicated the relation between extraversion and FRN reported previously by Smillie et al. (2011), in a subset of the current sample on whom we had extraversion data (*n* = 25). The size of this effect was comparable to that obtained by Smillie et al. (2011) in a sample of extreme high/low scorers on extraversion. The fact that almost no other trait examined in this study yielded a stronger association with our FRN RPE index offers considerable encouragement to reward-processing theories of extraversion (e.g., Depue and Collins, 1999).

Beyond trait extraversion, our key aim in the current study was to extend research in this area by examining a broader array of putatively BAS-related personality measures in relation to FRN. These measures encompassed a range of constructs that have previously or currently been considered as reflecting variation in the functioning of the BAS, and included measures of impulsivity, reward sensitivity and anhedonia. We were particularly interested in evaluating a relatively recent self-report measure, the TEPS (Gard et al., 2006), which has sought to dissociate the measurement of TEPS-ANT and TEPS-CON. We predicted that the FRN would significantly vary with TEPS-ANT, but not with TEPS-CON. Our findings supported this prediction; for those individuals higher on the TEPS-ANT scale, the RPE difference wave contrasting unpredicted non-reward and unpredicted reward trials was larger compared to those lower on TEPS-ANT. The TEPS-CON scale, on the other hand, did not significantly relate to FRN and this non-significant relationship was significantly weaker than that for the TEPS-ANT. We predicted that a similar dissociation would occur across the BAS scales in the Carver and White (1994) BIS/BAS scales, with positive correlations expected for BAS drive, but not for BAS reward responsiveness or BAS-fun-seeking. However our findings did not support this, with none of the BAS scales relating significantly to FRN. There were also no significant associations between the FRN and the measures of psychoticism and neuroticism from the EPQ-R, anhedonia (SHPS and BDI), and the BIS scale.

The findings from this study provide further support that variation in personality traits associated with behavioral approach, agency and anticipatory positive emotion are linked with FRN, a potential index of RPE signaling. The replication of our previous result with extraversion is particularly encouraging, given the relative inconsistencies in replication of effects linking extraversion with indices of reward processing (Smillie, 2013). While in both studies we used the EPQ-R measure of extraversion, it would be useful for further research to extend and validate this finding by examining lower order facets/aspects of extraversion, particularly those that potentially distinguish behavioral approach and enjoyment of rewards (e.g., agency vs. affiliation; Depue and Collins, 1999, assertiveness vs. enthusiasm; DeYoung et al., 2007). Our findings in relation to the TEPS are also encouraging and support the validity of the psychometric distinction between anticipatory and consummatory pleasure in this measure. While the TEPS has shown some initial promise, the validity of the distinction between anticipatory and consummatory pleasure remains to be further tested using reward processing paradigms.

None of the BAS scales from the Carver and White (1994) scales correlated with FRN in this study. This runs counter to some previous studies (Lange et al., 2012; Bress and Hajcak, 2013) that have found significant associations between scores on BAS scales and the magnitude of FRN. In one of these studies (Lange et al., 2012), however, an aggregated BAS scale score was used, and so it is unclear how the BAS subscales relate to FRN. We combined the three BAS scales in to a composite BAS score, as in Lange et al. (2012), however this aggregate BAS score also did not significantly relate to our RPE index, *r* = 0.13, *p* = 0.437. As outlined in the introduction, the association between the BAS subscales and the FRN RPE index was not expected to be significant for those subscales (reward responsiveness and fun-seeking), which emphasize reward liking in their item content. Items in these scales might index variation in the tendency to derive pleasure from obtained reward, rather than motivated behavior toward to-be-obtained reward (e.g., *When I get something I want, I feel excited and energized*; *When good things happen to me, it affects me strongly*). We predicted that the BAS drive subscale, which seems least characterized by such pleasure-focused, liking items, would correlate with our FRN RPE index. However, this subscale actually showed numerically the weakest correlation with the RPE index. Of note, the only BAS-relevant scale that BAS drive significantly correlated with in this study was the BAS fun-seeking scale; it did not correlate significantly with EPQ-R extraversion, nor with either of the TEPS scales.

In Bress and Hajcak (2013) a relatively new measure of reward responsiveness was used (Van den Berg et al., 2010). This new measure includes existing items from the Carver and White drive and reward responsiveness scales, and some novel items. The item content overall tends to reflect agency, drive, and anticipatory excitement. On that basis, the results from Bress and Hajcak (2013) are more consistent with the pattern of association we were predicting, while being somewhat at odds with our findings for the drive scale. Bress and Hajcak’s findings can perhaps be viewed as being broadly consistent with our findings linking FRN with agency and behavioral approach in EPQ-R extraversion, and anticipatory pleasure from the TEPS.

The results from this study should be considered in light of some potentially important limitations. Firstly, Cronbach’s α for the TEPS-CON scale was low (0.50), and this will have attenuated the correlation between this scale and the RPE index. The TEPS-ANT scale, which did show a significant association with the RPE index, did have an acceptable α-value (0.72) in this case. More generally, the TEPS is a relatively recently developed scale and so the psychometric properties of this scale clearly need further exploration. Beyond potential concerns with the reliability of the TEPS-CON scale, Ho et al. (in press) used confirmatory factor analytic modeling of the TEPS to show that while a two-factor structure best represented the data, model fit indices for a two-factor model were less than adequate; this may have been at least partly driven by cross-loading of items across each scale, as has also been shown in other previous studies of this measure (e.g., Gard et al., 2006). Given the importance of having a well-validated and reliable self-report measure that dissociates anticipatory and consummatory reward processes, further research and development on the TEPS should be encouraged. It may be that some modification of this measure is required moving forward. Similarly, the anhedonia subscale from the BDI also had very low reliability in this sample, and so the non-significant relationship between this scale and the RPE index may be explained on this basis.

Another potential limitation in the study is the use of a passive associative learning task. Given that participants are not required to make any behavioral responses to the task, it may be that confounding variables related to the passive nature of the task, such as attention or boredom-proneness, became important. It should be noted, however, that the personality variables used in this study were not significantly correlated with the number of trials removed because of movement and other artifacts; these artifacts may partly reflect variables such as lack of attention. Future studies might benefit, however, from using a modified task that includes mixed blocks of active and passive responses to the task contingencies. Indeed, Martin and Potts (2011), using a similar task to that used in the current study, alternated passive and active response blocks of trials in the task. They showed significantly enhanced FRN to outcomes that were worse than expected only in the active condition, although there was a non-significant trend in this direction in their passive condition. If a more robust FRN effect is reliably obtained using active responses, then it may be more useful to study personality-based individual differences using FRN tasks that involve an active response. More generally, self-reported level of task interest and engagement are higher in response vs. no response tasks, and the difference in task interest between response vs. no response versions of tasks correlates with FRN magnitude (Yeung et al., 2005), so it would be useful for future personality research in this area to assess task engagement, subjective reward expectation and level of attention more systematically.

This study adds to the literature showing that FRN, as a putative marker of RPE signaling in brain dopaminergic “reward” pathways, is related to scores on self-report personality measures. More specifically, we replicated our previous result (Smillie et al., 2011) showing that trait extraversion, as measured using the EPQ-R and characterized by a focus on behavioral approach and agency, was significantly related to this RPE index. We also showed that the RPE index correlated significantly with the TEPS measure of anticipatory pleasure, but not consummatory pleasure. This provides support for the notion that individual differences specifically in behavioral approach and anticipatory positive affective states are at least partly underpinned by functional variation in dopaminergic systems. This finding might partly be qualified by a lack of dissociation in associations with FRN across the three BAS subscales in the Carver and White (1994) BIS/BAS scales. Nonetheless, it is hoped these findings further contribute to an understanding of how broad-level personality traits, like extraversion, relate to neural responses to rewarding events. In that respect, we also hope these findings provide encouragement for further work examining the separable role that anticipatory and consummatory reward processes may play in personality structure and processes (Smillie, 2013).

## Conflict of interest statement

The authors declare that the research was conducted in the absence of any commercial or financial relationships that could be construed as a potential conflict of interest.
